# A Key Role for the Genetic Counsellor in the Genomics Era

**DOI:** 10.12688/f1000research.14222.2

**Published:** 2018-12-17

**Authors:** Flora M. Joseph

**Affiliations:** 1 All Wales Medical Genetics Service, Cardiff, UK

**Keywords:** genetic counseling, genetic services, genetic testing, intellectual disability, developmental disabilities, psychosocial support systems, uncertainty, psychological adaptation

## Abstract

New genetic testing technologies such as microarrays and whole exome sequencing mean the diagnostic potential for a child with a development disorder is greatly increased over traditional testing techniques.  With this increased potential comes increased expectations from families and professionals about the answers a diagnosis will provide.  However, limitations remain and a proportion of individuals will continue to remain undiagnosed.  In addition, some individuals will receive novel or very rare diagnoses about which very little is known in terms of prognosis and effective treatments.  In this paper, I present an argument for why these families would benefit from additional Genetic Counsellor support and how Clinical Genetics services in the UK could provide this support.  I acknowledge that resources are limited, but as demands on services increase and interactions with families become shorter, I argue that this kind of service should be prioritised, for the benefit of these families.

## Introduction

Projects such as the Deciphering Developmental Disorders (DDD) study, and the 100,000 Genomes project, have used whole exome or genome sequencing to pinpoint disease causing mutations (Firth & Wright, 2011; Genomics England, 2018), increasing the chance of making a diagnosis in individuals with developmental disorders. This type of technology is a realistic possibility for clinical NHS care in the UK within the not-too-distant future ( Hazelton & Petchey, 2015). In this paper, I explore the implications this could have for the support needs of families of children with developmental disorders as they go through the diagnostic journey. I discuss the role that Genetic Counsellors can play in meeting these support needs and argue that extending the role in the post-testing stage would benefit these families.

Developmental disorders may manifest in any area of development (e.g. growth, congenital malformations, seizures) but the most common phenotype is developmental delay (DD) or intellectual disability (ID) ( Deciphering Developmental Disorders Study, 2015) which are common referral indicators to Clinical Genetic Services. DD may present itself in any of the following areas: gross/fine motor skills, speech/language, cognition, social/personal, and activities of daily living ( Shavell *et al.*, 2003). If a child has delay in two or more of these areas they are said to have Global Developmental Delay (GDD). The estimated prevalence of GDD is about the same as ID at approximately 1–3%. It is estimated that 5–10% of all children have some sort of DD, therefore DD or even GDD may not necessarily lead to ID, but children with ID often had DD in their early years ( Shavell *et al.*, 2003).

## Diagnostic tools employed for individuals with developmental disorders

Traditionally, those fulfilling the clinical features of a recognised syndrome may be diagnosed on clinical examination alone. As technology has advanced, other diagnostic tools have become available, such as metabolic studies, EEG, CT/MRI imaging, cytogenetic studies (e.g. karyotype, FISH studies, subtelomeric studies, etc), and single-gene targeted testing ( Rauch *et al.*, 2006; Shavell *et al.*, 2003). Until the advent of more recent technologies (array CGH and next generation sequencing), these tools gave a diagnostic yield for children with GDD/ID in the region of 50–70% ( Daily *et al.*, 2000; Rauch *et al.*, 2006). Therefore, up to half of children with GDD/ID remained undiagnosed.

‘Molecular karyotyping’ or ‘array Comparative Genomic Hybridisation’ (array CGH) is a technique that came into clinical practice across the UK during the first decade of the 21st century ( Rauch *et al.*, 2006). Array CGH identifies sub-microscopic genetic imbalances across the genome. It can identify copy number variants (changes in the number of copies) of genes which may affect health or development. It allows much more detailed cytogenetic analysis and in most parts of the UK has become a first-line test, superseding traditional cytogenetic studies. Array CGH gives an average diagnostic yield of 15–20% ( Miller *et al.*, 2010) for individuals who have not received a diagnosis using other methods. As our knowledge advances, it is hoped the classification of variants of uncertain significance will improve, which could increase this diagnostic yield.

The Deciphering Developmental Disorders (DDD) study was a UK-based study sequencing the exomes of children with undiagnosed developmental disorders (Firth & Wright, 2011). Exomes are the coding regions of our genes, and account for about 1% of all our genetic material ( Wang *et al.*, 2013). The DDD study hoped that a molecular diagnosis could be found by comparison of a child’s exome with their parents’ (Firth & Wright, 2011). A diagnostic yield of 31% was reported from the first 1133 trios (children and both parents) recruited to the study, and 12 new developmental genes were discovered ( Deciphering Developmental Disorders Study, 2015). An American-based study sequencing clinical exomes also found a diagnostic yield of 31% for trios, and 22% for proband-only cases ( Lee *et al.*, 2014). The diagnostic yield is expected to increase as new genes are described and further analysis takes place.

## Responses and support needs of parents adapting to having a child with a developmental disorder

It is natural for parents to hope for a healthy child. The point when a developmental disorder may become apparent can range from a prenatal scan, to birth, to later on as the child grows. This can be a sudden or a gradual realisation ( Heiman, 2002; Lewis *et al.*, 2010). Initial reactions to this realisation are likely to be negative such as disbelief, anger, denial, and grief ( Graungaard & Skov, 2007; Heiman, 2002; Kearney & Griffin, 2001; Lewis *et al.*, 2010). From this initial reaction, parents must go through a process of adaptation of “replacing the hopes and expectations…with the realities of their child’s actual prognosis” (pg 186, Barnett *et al.*, 2003). This process has been likened to that of a ‘journey’ that at times is like a ‘rollercoaster’ full of highs and lows ( Lewis *et al.*, 2010).

For parents to adjust appropriately to the reality of having a disabled child, they must adopt a number of coping strategies. These may be practically or emotionally-directed ( Graungaard & Skov, 2007; Lewis *et al.*, 2010; Rosenthal *et al.*, 2001). Practical coping strategies may be information gathering e.g. about disease prognosis, treatments or research opportunities. Emotional coping strategies may be talking with friends and family or accessing support services. Some of the studies cited earlier went on to explore parental feelings after they have gone through this period of adaptation. They found that many parents have positive and optimistic feelings about their child’s future ( Heiman, 2002; Kearney & Griffin, 2001; Lewis *et al.*, 2010).

Although the majority of parents appear to adapt well to their new reality, some parents and families are less successful. Traits of less successful adaptation may be: unrealistic appreciation of their child’s weaknesses and limitations; continued feelings of self-pity and guilt; searching for a ‘magical solution’; and feelings of rejection or over-protection of their child, sometimes at the expense of other family members ( Kandel & Merrick, 2007). This inability to cope and adapt can have a negative effect on mental health (e.g. stress, depression), relationships and functioning ( Barnett *et al.*, 2003).

A developmental disorder can leave families with many questions, some of which can only be answered by obtaining an accurate diagnosis. In this way, searching for a diagnosis can form part of the process of coping and adaptation. Below is a list of reasons given for seeking a diagnosis that has been amalgamated from a range of studies. Most of these studies are of parents of children with developmental disorders, but one includes responses from adults with genetic disorders ( Hazelton & Petchey, 2015). A range of methods were used including semi-structured interviews ( Lewis *et al.*, 2010; Makela *et al.*, 2009; Rosenthal *et al.*, 2001) and surveys ( Hazelton & Petchey, 2015; Limb *et al.*, 2010; Madeo *et al.*, 2012).

- To provide information about progression and prognosis for their child to make life plans and help form realistic expectations for the future
- To be aware of recurrence risk for future pregnancies and whether pre-natal testing or carrier testing is available for relatives
- To guide clinical management e.g. whether any additional clinical surveillance is recommended or whether any treatments, therapies or diets are known to be ineffective/harmful for that condition
- To have ‘a label’. Both positive and negative associations were attributed to this. For example, a benefit is having a term to explain why their child is different from other children. However, a concern is that it may cause their child to be stereotyped and people, such as teachers, to have reduced expectations of their abilities
- To improve access to support services (e.g. education, health or social services). Although this should be based on need regardless of diagnosis, this has been reported to be easier when a diagnosis is known ( Rosenthal *et al.*, 2001)
- To improve access to peer support of families in a similar situation as themselves. Without a diagnosis, there can be increased feelings of isolation
- To have ‘an answer’. This can provide psychological relief to keep from wondering why this has happened and increase perceived feelings of control

Rosenthal *et al.* (2001) and Lewis *et al.* (2010) each conducted semi-structured interviews with parents of children with developmental disorders either in the USA (Rosenthal group) or the UK (Lewis group). The purpose of these studies was to find out what impact a lack of diagnosis had on parental adjustment and coping. There was a wide range in the length of time that parents had been aware of their child’s difficulties in both studies, which provides useful information about how coping and adaptation may change over time. Similar reactions to the initial recognition of a problem were reported by parents whether a child had a known diagnosis or not ( Lewis *et al.*, 2010). However, in the absence of a diagnosis, additional challenges were noted which could result in a longer period of adjustment and adaptation ( Barnett *et al.*, 2003; Heiman, 2002; Lewis *et al.*, 2010; Rosenthal *et al.*, 2001).

A lack of diagnosis means there is greater uncertainty. Madeo *et al.* (2012) looked at the effect of uncertainty on parental coping and adaptation to raising a child with a developmental disorder. Lipinski *et al.* (2006) also looked at factors associated with parental uncertainty and perceived control, and the role they played in coping and adaptation for parents of children with a rare chromosome disorder (prevalence of 1/120,000 or lower), which provides useful information about families with novel or very rare conditions. Both studies used a mixed-methods survey (either paper or computer-based) and had relatively large sample sizes (266 and 363 respectively). Madeo *et al.* (2012) discussed that uncertainty can sometimes aid coping, as it leaves room for optimism of a positive outcome. However, in the majority of cases, both studies found that uncertainty perpetuated a feeling of lacking control over their child’s condition, which was linked to poorer coping and a longer process of adaptation, as reported by Rosenthal *et al.* (2001) and Lewis *et al.* (2010). Both Lipinski *et al.* (2006) and Madeo *et al.* (2012) found factors that were associated with lower perceived control were being less optimistic about the future and perceiving their child’s condition as more severe. Lipinski *et al.* (2006) found younger parents felt greater uncertainty but this was not replicated by Madeo *et al.* (2012), although the latter study population did not have a very wide age range. These studies help to explain why a lack of diagnosis, or the diagnosis of a very rare or novel condition, often results in an extended period of coping and adaptation and therefore why additional support may be appropriate.

As one parent from the Genetic Alliance UK patient charter commented “ *We always imagined that getting a diagnosis would be the final piece of the puzzle and the end of the journey, but it now feels as if we are at the very beginning of a new journey*” (pg 9, Hazelton & Petchey, 2015). Another parent, whose child was found to have a unique unbalanced translocation, used the term ‘non-diagnosis’ as there was no prognostic information available and commented “ *It’s like being told something in a foreign language. It wasn’t a relief because I didn’t understand it*” (pg 810, Lewis *et al.*, 2010). For these parents the diagnosis had not brought all the answers they had hoped for.

Parents described how the desire for a diagnosis diminished over time, as the child grew older, however often it never completely went away ( Lewis *et al.*, 2010; Rosenthal *et al.*, 2001). In part, this may be due to becoming more familiar with their child’s condition as they grow, and forming a clearer idea of what the future may be like. It addition, this may be due to the realisation that a diagnosis will not change who their child is. However, there are times that re-kindle the desire for a diagnosis e.g. when the child is approaching adulthood and applying for additional support (e.g. supported housing), or when siblings are reaching reproductive age, and additional support could also be appropriate at these times.

As genetic testing becomes more accessible and diagnostic rates increase, I wonder how this will affect the support needs of families. Even though diagnostic rates will improve, there will undoubtedly be individuals who remain undiagnosed. In addition, there will be a growing number of individuals who are given a novel molecular diagnosis, about which very little may be known. While these families may receive answers to some of their questions, others will remain unanswered. I wonder whether the current role of the Genetic Counsellor is sufficient to meet the needs of these families, or whether adaptations to the role could meet the need better.

## The role of the Genetic Counsellor

Biesecker (pg 327, 2001) discussed the goals of genetic counselling and stated that “contemporary genetic counselling should strive to… facilitate clients’ ability to use genetic information in a personally meaningful way that minimises psychological distress and increases personal control”. Genetic Counselling can be provided by clinicians with specialist training in genetics. Traditionally this has predominantly been Clinical Geneticists and Genetic Counsellors/Genetic Nurse Specialists. As genetic testing becomes more mainstreamed this has broadening to include a wider range of clinicians such as Paediatricians, Oncologists, Cardiologists, Obstetricians and Specialist Nurses working in these areas ( Middleton *et al.*, 2015; Welsh Government, 2017). In the UK, Genetic Counsellors work alongside Clinical Geneticists and Clinical Scientists. Whereas other specialities may have detailed discussions about a test result, and be empathetic with a patient, they are unlikely to discuss familial implications and have capacity to have lengthy discussions about emotional impact and processing, so Genetic Counsellors have the capacity for more detailed discussion and exploration of these implications ( Middleton *et al.*, 2015).

Current training programmes in the UK are through a Masters programme, 2 or 3 years in length, which includes training in the science of genomics, counselling theory and practical experience in Regional Genetics centres ( AGNC, 2018). The current training programmes started in 2017 in response to the move towards more genomic testing and include a larger focus on interpretation of genomic results. Therefore, Genetic Counsellors trained prior to this may feel the need for further training in genomic testing, result interpretation and the limitations of advanced genetic testing technologies in order to appropriately counsel families.

Families are for the most part resilient, but the process of coping and adaptation still benefits from psychological support, regardless of whether a child receives a diagnosis or not ( Barnett *et al.*, 2003; Lewis *et al.*, 2010). Seymour Kessler described two models of practice for Genetic Counsellors, a ‘teaching model’ and a ‘counselling model’ ( Kessler, 1997). Kessler suggests that a hybrid approach is adopted incorporating elements of both so that the counselee has received the appropriate information but has also had time and space for discussion of the consequences and personal reflection. However, a number of studies have examined modern practice and found that the ‘teaching model’ is more often adopted, with the main purpose of information provision ( Lerner *et al.*, 2014; Meiser *et al.*, 2008; Roter *et al.*, 2006; Walser *et al.*, 2017). Austin *et al.* (2014) reviewed the available literature and found that a ‘counselling model’ with the aim of addressing the psychosocial concerns, is reported to be associated with increased knowledge retention, reduced anxiety and higher satisfaction with decision-related outcomes. The authors go on to suggest that Genetic Counsellors focus more on this style in their practice. This is supported by Lipinski *et al.* (2006) who looked at the perceived helpfulness of genetic counselling, for parents of children with developmental disorders. The authors found that it was perceived as more helpful when parents were helped to increase a sense of perceived control over their child’s condition ( Lipinski *et al.*, 2006) which fits more with a ‘counselling model’ of practice. A study from the USA looking at the result-giving appointment of exome sequencing highlighted that these appointments were information-heavy and often missed opportunities to build relationships with patients ( Walser *et al.*, 2017). The counselling element tended to be neglected due to time restraints and so an on-going relationship with these families would be particular important, to improve understanding, reduce misconceptions, address frustration and disappointment, and improve satisfaction.

Genetic Counsellors have historically had the resources to offer long-term support to families in the UK. Co-counselling with Clinical Geneticists, where they would see patients together, also use to be more common. However, as demands on Genetic Services have increased, time restraints have limited the amount of contact Genetic Counsellors are able to offer families, and relationships have become shorter-term and co-counselling has reduced. Therefore, even though the need for support still exists, families may not request it as they may not recognise where this form of support is best sought ( Lipinski *et al.*, 2006). Genetic testing and genetic understanding is infiltrating many areas of healthcare, and the role of Genetic Counsellors as ‘information providers’ is becoming less specialised ( Austin *et al.*, 2014) however Genetic Counsellors have a unique set of skills and can play an important role in providing psychological support for families ( Lipinski *et al.*, 2006; Middleton *et al.*, 2017). Austin *et al.* (2014) propose genetic counselling is remodelled “as a time-limited, highly circumscribed psychotherapeutic encounter”. Whereas there is some support for this ( Wynn, 2016) , from my own experience I feel there is doubt about whether this sort of service is viable in the UK at the moment, due to increased demands and limited resources. I would like to see the profession working towards this sort of model for families of children with developmental disorders and I describe below how this could look in practice.

## Implications for Genetic Counselling practice

Genetic testing is becoming more mainstreamed, in some cases without Clinical Genetics involvement. In addition, due to increases in referrals to Clinical Genetics, initial information gathering from families (which has historically been the role of the Genetic Counsellor and an opportunity to assess how families are coping) varies between regional Genetics Services in the UK and is sometimes being carried out by family history coordinators or by postal questionnaires. Therefore the opportunity for pre-test counselling by a specialist in Genetics is reducing. With the vast amount of information that can be generated from genomic testing, I see the real value of the Genetic Counsellor’s role being in the post-testing period, where the emotional and familial context can be explored ( Middleton *et al.*, 2015). Whereas there is already often an open-door policy for families to have follow-up appointments in many genetics services, families may not recognise where this support is best sought, as mentioned above ( Lipinski *et al.*, 2006). I propose that for some families, it may be beneficial to build in routine post-test follow up to the patient pathway. As many Genetic Services are already under great pressure with current demands, adding this additional service may not currently be viable. However, the need for extra funding into genomics in healthcare is being recognised as demonstrated in the Welsh Government’s ‘Genomics for Precision Medicine Strategy’ (2017). This describes the anticipated increase in genomic testing and the increased demand this will put on clinical genetics services. It states that funding will be needed to increase the clinical genetics workforce and for the increased training needs of genetic and non-genetic staff. In addition, it describes how patient experiences will drive service improvements and recognises that Clinical Geneticists and Genetic Counsellors are key elements in providing a good patient experience. Therefore I would hope that the kind of service I describe could be included in future planning.

### Proposed aims for a post-test Genetic Counsellor clinic

For families identified as needing additional support, a time-limited intervention (e.g. 1-3 Genetic Counsellor consultations) could be offered. The aims would be to minimise psychological distress, increase personal control and either i) help them cope with an uncertain future in the absence of a diagnosis, or ii) readjust to having a diagnosis for their child, including coping with the uncertainties this brings if this is for a very rare or novel condition. 

Whereas all families may benefit from extra support, I feel some families would benefit in particular, as I describe below. 

### Identifying families in need

Once all available investigations have been exhausted, or if results are not expected for a very long time (e.g. via a research study), patients could be considered for the post-test Genetic Counsellor clinic. Indicators for additional support may be:

- The family have lower levels of perceived control/increased levels of uncertainty
- The family have a lack of social support
- The affected person has a novel or very rare diagnosis about which little information is known
- The affected person remains undiagnosed

***Assessing perceived control.*** Increasing personal control over the situation aids coping and adaption ( Biesecker, 2001; Lipinski *et al.*, 2006; Madeo *et al.*, 2012). As Lipinski *et al.* (2006) and Madeo *et al.* (2012) found, lower perceived control is associated with being less optimistic about the future and perceiving their child’s condition as more severe. Therefore, families with these indications may benefit from additional support. One suggestion is to use the Revised Life Orientation Test (LOT-R) which rates 10 statements to assess optimism ( Madeo *et al.*, 2012).

When there are high levels of uncertainty, emotional-focused coping strategies are often required to increase perceived control over the situation ( Lipinski *et al.*, 2006). The Negative Mood Regulation (NMR) scale is a 30 point scale that measures a person’s belief in their own emotion-focused coping resources and may be a helpful way of identifying families in need ( Catanzaro & Mearns, 1990). Asking families about their child’s abilities compared to healthy children of the same age might give an indication of their perceptions of the severity of the child’s condition and if there is a great difference between the parents’ and the clinician’s perceptions, further clarification may reduce perceived uncertainty ( Madeo *et al.*, 2012).

Another indicator may be the time elapsed since recognition that their child has a developmental disorder. Rosenthal *et al.* (2001) and Lewis *et al.* (2010) found the desire for finding a diagnosis decreased with time. Therefore, families nearer the beginning of their journey may benefit more from additional Genetic Counsellor support ( Lipinski *et al.*, 2006).

***Assessing sources of social support.*** By asking what sources of support the family have around them, or who they talk to about their child, families may be identified that are experiencing isolation and are in greater need of additional intervention. To effectively highlight families in need, a proforma could be designed that asks for the indicators specified that could be completed by the relevant Clinician (Clinical Geneticist or Genetic Counsellor) to refer the family for the post-test Genetic Counsellor clinic. Alternatively a questionnaire could be included with a post-clinic letter, asking questions around perceived control, optimism and support, to be returned should the family wish to engage with further support.

### Proposed model for a post-test Genetic Counsellor clinic

The objectives of the session could be:

- Elicit and address concerns
- Discuss strategies for increasing perceived control
- Signpost to other sources of support

***Eliciting and Addressing concerns.*** For families of children with a developmental disorder, it may be that some of their concerns are hindering their ability to adapt to their child’s condition (Graungaard & Skov, 2006; Rosenthal *et al.*, 2001). Families who place a greater significance on finding a diagnosis may struggle more if one is not made. However, by exploring their reasons for seeking a diagnosis, the Genetic Counsellor may be able to help them find resolution to their concerns, even in the absence of a diagnosis, for example, by accessing peer-support.

Another concern that could be discussed in the absence of a diagnosis, is having a term or label for their child to use with other professionals, or with friends and family e.g. ‘developmental delay’, ‘a SWAN child’ (after the support group Syndromes Without A Name) ( Lewis *et al.*, 2010). The Genetic Counsellor, in partnership with the Clinical Geneticist, could help families to come up with terminology they can use.

***Discussing strategies for increasing perceived control.*** For the questions that cannot be answered (e.g. prognosis or recurrence risk) families will continue to have a degree of uncertainty, which may perpetuate a feeling of lacking control. As Lipinski *et al.* (2006) found, genetic counselling was perceived as more helpful when parents are helped to increase a sense of perceived control over their child’s condition. Madeo *et al.* (2012) suggests Genetic Counsellors may help families by identifying areas where they do have some control. The authors suggest training in interventions, such as Coping Effectiveness Training “in which individuals identify the controllable and uncontrollable aspects of their situation and are assisted in identifying coping strategies that are predicted to best match the controllability of the stressor” ( Madeo *et al.*, 2012).

Practical-focussed strategies, such as information gathering, may give an increased perception of control ( Madeo *et al.*, 2012). Lewis *et al.* (2010) provides some other suggestions given during interviews with parents. Examples of these are ( Lewis *et al.*, 2010; Madeo *et al.*, 2012):

- Keeping a diary to monitor their child’s condition and progress. This will not only serve as a useful tool when talking to health care providers, but can also act as a reminder of the progress their child has made
- Developing ‘a passport’ of their child’s likes/dislikes, what they can/can’t do, and their medical problems can aid communication with professionals
- Learning about medicine and treatments that are being offered for their child. In this way parents can feel they are making more informed decisions about management
- Becoming experts and advocates for their child’s condition. Rosenthal *et al.* (2001) found that parents who felt informed about their child’s problems felt empowered to act as advocates for them in obtaining support services (e.g. educational).
- Contributing to fundraising or research for their child’s condition or for rare diseases in general. Rosenthal *et al.* (2001) found a keenness from parents to engage in activities such as these, so that their child’s disability may benefit others
- Setting up a blog or website about their child. This can be both therapeutic and create opportunities for networking and advocacy

However, when there is a high level of uncertainty, practical coping strategies may not be successful and may reduce perceived control due to a lack of available information. In these instances, emotion-focussed strategies may increase perceived feelings of control as “one’s internal state may be more amenable to change than the situation itself” (pg 239, Lipinski *et al.*, 2006). These coping strategies may be talking with friends and family, accessing support services, retaining hope and focussing on the positives. Lipinski *et al.* (2006) reported that parents would have liked Genetic Counsellors to have more hope and encouragement. This is not limited to hope that a diagnosis will be made, but also hope for the future, even in the absence of a diagnosis (e.g. in what support their child may obtain and hope for prognosis) ( Graungaard & Skov, 2007).

***Signposting to other sources of support.*** Feelings of isolation and not knowing where to turn for support were often cited as reasons for seeking a diagnosis ( Lewis *et al.*, 2010; Rosenthal *et al.*, 2001). Parents report a lack of information about what educational, social and psychological help is available ( Heiman, 2002). If a Genetic Counsellor is aware of the local groups and resources available, these can be provided. Resources could be provided that contain a broad range of information, such as the roles of different healthcare providers, about educational support and benefits for children with developmental disorders. For example, the support group Unique ( www.rarechromo.org) has a useful booklet called ‘After diagnosis: What happens next? The early years’.

Support groups can provide peer-support for families in a similar situation. In the UK, the support group ‘Syndromes Without A Name (SWAN) UK’ ( www.undiagnosed.org.uk) has been formed specifically for families of children without a diagnosis.

Support groups can also be a useful source of information about relevant research projects ( Limb *et al.*, 2010). Patients report that they rarely hear of research opportunities relating to their condition from clinicians ( Limb *et al.*, 2010). This is understandable to an extent as it is challenging for clinicians to stay abreast of all relevant opportunities when dealing with multiple conditions. Therefore, this should be highlighted to patients as a benefit of being part of a support group. Many families who were recruited to the DDD study first heard of it through SWAN UK ( Hazelton & Petchey, 2015).

For families affected by very rare or novel conditions, such groups may not exist and feelings of isolation can persist ( Rosenthal *et al.*, 2001). In the UK, some more general support groups exist such as Rare Disease UK ( www.raredisease.org.uk), and Unique ( www.rarechromo.org). Unique was originally set up for families affected by rare chromosomal abnormalities. However, they have now broadened their spectrum to include families affected by very rare autosomal dominant single gene disorders, where a more specific support group does not yet exist ( Unique, 2018). For these families, the challenges can be very similar to those with rare chromosomal conditions and the peer-support reduces feelings of isolation, which can be immensely empowering ( Limb *et al.*, 2010).

In time it is likely that new information and/or tests will become available as research continues ( Lipinski *et al.*, 2006). It would be important to emphasize to families of the option of a review appointment in Clinical Genetics if any new symptoms or features arise; when questions arise again such as the child reaching adulthood and possibly leaving home; or when siblings reach reproductive age. This will also hopefully lessen any feelings of abandonment.

## Conclusion

In the age of advanced genetic technologies, expectations have never been higher about the diagnostic potential for individuals with developmental disorders. However, a diagnosis will not necessarily provide all of the answers sought. In addition, the search for a diagnosis could be hindering a family’s ability to accept their child’s condition. Without appropriate support, the process of coping and adaptation could be prolonged or even unsuccessful. Genetic Counsellors have the skills to provide the support required to address the uncertainties families face, help identify areas where peer support can be found and increase perceived control, facilitating adaptation to improve individual and family functioning. This service could be provided in a specific post-test clinic to which families could be referred. Whilst Genetic Services in the UK may currently not have the resources to facilitate such an extended support service due to current demands, Genomics is an area of great development and now is the time to ensure the role of the Genetic Counsellor is optimised for these families. I believe this type of service is the very essence upon which the Genetic Counselling profession was built and it is vitally important for these families that this support is integrated into genomic testing so their psychosocial needs are not neglected in the flood of genomic information.
