# Supplementary material for: A Key Role for the Genetic Counsellor in the Genomics Era
Source: F1000Res. 2018 Dec 17;7:420. Originally published 2018 Apr 3. [Version 2] doi: 10.12688/f1000research.14222.2 (PMC6338239; doi:10.12688/f1000research.14222.2)
Supplement: Peer review history

# Peer review: Referee response for version 2

**DOI:** 10.5256/f1000research.18833.r42020

**Published:** 2019-01-17

**Authors:** Charlotta Ingvoldstad Malmgren

**Affiliations:** 1 Department of Public Health and Caring Sciences, Uppsala University, Uppsala, Sweden; 2 Clintec, Karolinska University Hospital, Stockholm, Sweden

I think it is much better structured, and easily read now. I also think it is good that the manuscript now focuses more on the post-test counselling and I really see the value of genetic counsellors in this process.

 I think this aspect of counselling is very important and I am happy that the author highlights this area of work setting for genetic counsellors and hope that clinical genetic clinics can benefit from this article in building their organisations.

 After the revisions made I now approve the manuscript for indexing in F1000.

I have read this submission. I believe that I have an appropriate level of expertise to confirm that it is of an acceptable scientific standard.

# Peer review: Referee response for version 2

**DOI:** 10.5256/f1000research.18833.r42021

**Published:** 2019-01-11

**Authors:** Andrew Cuthbert

**Affiliations:** 1 Cardiff University, Cardiff, UK

Very much improved, thank you!

 Although not relevant to reviewing this manuscript, I feel there is a debate to be had about the relative merits of genetic counsellors as opposed to other disability oriented health professionals (who see many more disabled children and their families with both acquired and genetically driven disabilities), third sector support groups, virtual/online/interactive resources, others, to best support families who are considered to be in need. A strong focus on health and social outcomes would have a priority. Perhaps, something a well designed research study could investigate? It would also be helpful to understand what hard-pressed parents (the ones who rarely engage in research!) actually want, what are their priorities. Addressing their social circumstances by other means, through education (a generational issue) and social care (more immediate) might improve their self-perception and sense of control over these difficult matters, and, vitally, their children's developmental outcomes.

 A few minor comments:

 1.  A formal definition of ID in the intro (alongside the given definition of DD) would be valuable.

 2.  Diagnostic yields achieved 50-70% WITH (not UNTIL) the advent of arrays and sequencing.

 3.  "...often results in an extended period of coping and adaptation...", but surely the coping is life long? Suggest  say "protracted period for acceptance and adaptation".

 'Referral criteria':

- The family have lower levels of perceived control/increased levels of uncertainty - **How would this be formally and equitably assessed? Is the LOT-R validated for perceived control? It was designed more to assess optimism vs pessimism, is this a valid surrogate of personal control?**
- The family have a lack of social support - **Designing a fair and equitable disability oriented social deprivation risk assessment tool would be enormously challenging, history shows.**
- The affected person has a novel or very rare diagnosis about which little information is known - **very** **difficult to equitably delineate this. Rare does not equate with complexity of care, extent of disability, impact on family. Consider the huge variation in presentation children diagnosed with the same CNV syndrome.**
- The affected person remains undiagnosed. - **S** **hould always be in follow-up and/or a research study.**

4. Generally, try not to cite the same reference multiple times, ie Lipinski 2006, Madeo 2012, Middleton 2015. Use other sources.

I have read this submission. I believe that I have an appropriate level of expertise to confirm that it is of an acceptable scientific standard.

# Peer review: Referee response for version 1

**DOI:** 10.5256/f1000research.15473.r34842

**Published:** 2018-07-09

**Authors:** Andrew Cuthbert

**Affiliations:** 1 Cardiff University, Cardiff, UK

**Title**

 Too long and tentative, be succinct and give a definite message to attract the reader.

**Aim**

 Needs a clearer definition of the aims. Is it to define the challenges faced by parents of children with DDs, diagnosed or otherwise, as well as the how well genetic counsellors address these issues? Key aim is a justification for extending genetic counsellor’s role in the post genomic diagnosis period, make this very clear early on. Needs to consider and compere alternative models of family support.

**Structure**

 Somewhat confused, needs clearly define and describe each topic and refrain from commentary therein, rather to have clearly subtitled commentary paragraphs where appropriate, either as a standalone section or at the end of each topic, and be consistent.

**Introduction**

 Unnecessary detail on definitions of DD and ID, some of which is inaccurate. Should think about the audience who may not be familiar with jargon such as exome sequencing. It would help to describe typical referral pathways to clinical genetics, most referral for DD/ID are from paediatric neurology services who frequently request array tests and return results prior to visiting genetics. How will this change, if at all, as genome sequencing is commissioned into the NHS. IS there a different perspective in the US, Europe, elsewhere?

 The description of diagnostic measures and yields is misleading. The focus ought, to be, briefly, on the yield (actually 15-23%) of current methods in use in UK services (or wherever the author feels this is relevant). Clinical microarray was originally commissioned in around 2007 (NHS England), ie it’s not so modern. Then should describe potential impacts of integrating exome/genome sequencing with increased genomic diagnoses (around 70%) and how this will impact current GC practice. 

 Needs a paragraph on the psychosocial imperatives of a genetic diagnosis/non-diagnosis and the role of GCs (or others) in counselling for this. Following this present a concluding paragraph outlining the commentary’s aims and objectives – and why there is, arguably, any need to change current practice.

**Genetic Counsellors and Genetic Counselling**

 Genetic counsellors are not the sole providers of genetic counselling (needs also to acknowledge clinical geneticists have been counselling families for as long as GCs). The commentary should be cognisant of the role of nurse specialists who provide counselling, ie that genetic counselling is not the exclusive the domain of trained GCs.

 Since developments in genome technology are inevitably influencing developments in service design and delivery, in the UK and other countries, the author should consider their impact on GC roles in supporting families post-diagnosis - as pre-test counselling becomes increasingly obsolete as has been happening for several years due primarily to technological developments and their superior diagnostic accuracy. Argue whether any change in service delivery might be justifiable.

**Support**

 The author should recognise and comment that a majority, probably vast majority, of families engage support from places other than clinical genetics, especially over the medium-long term. Genetics services role in supporting families is circumscribed and predominantly short-term.

 The nature of support is vaguely described, what exactly is the support given in a genetic counselling encounter (by a genetic counsellor or other practitioner) which would facilitate improved adjustment and where is the justification to develop a specialist post-test/post-genomic diagnosis support service as distinct from routine follow-up. A significant Would this be a genetic counsellor led service? How would this differ from current open-door policy where parents are able to access follow-up contact with the clinician/practitioner. The author could consider how signposting and information content and provision could be improved, particularly how to ensure support group contact is increased, training for rare disease support groups to tackle complexities associated with single gene diagnoses for DD/ID after exome/genome sequencing, as with 100k genomes. Arguably, well informed peer/third sector support is more accessible, immediate and broader in relevance than highly specialised genetic counsellors/clinical specialists and perhaps better placed to facilitate longer term ‘community’ support, family acceptance and adjustment.

 Psychological support for families with children with severe/life limiting illness, more generally, isn’t dependent on the disease specialist being trained to provide such additional ‘support/care’. Arguably this applies to aspects of rare disorders care ad management. Moreover, given the extent and complexity of mental health risks, impacts and needs of children with DD/ID, combined with the psychological and social struggles of their parents/carers, who experience high levels of anxiety, depression and trauma (as the author acknowledges), are there alternative models of ‘support’ which are more rigorous and wide-ranging than conventional genetics follow-up, for which mental health and voluntary sector providers would be better placed to provide? A clear definition of the purpose, content and contextual relevance of ‘support’ needs to be given, as forms the basis of the authors justification for a significant change in service.

**Conclusion**

 This is a thought provoking commentary on a subject of increasing importance and at a time when genomic medicine’s relevance and promise to improve lives is subject to intense scrutiny, presented at a time when certain aspects clinical genetics services are experiencing perceived existential threats. Despite its shortcomings, need for structural correction and lack of coherent justification for what would be a significant change to current service provision which is counter to the evolving emphasis of genetic counselling training programmes, this kind of commentary is welcome and strongly supported. A revised version would be very welcome.

I have read this submission. I believe that I have an appropriate level of expertise to confirm that it is of an acceptable scientific standard, however I have significant reservations, as outlined above.

# Peer review: Referee response for version 1

**DOI:** 10.5256/f1000research.15473.r34843

**Published:** 2018-06-28

**Authors:** Sue Kenwrick, Vicki Wiles

**Affiliations:** 1 Addenbrookes Hospital, Cambridge, UK

**Comments by Sue Kenwrick, Principal Genetic Counsellor**

 The author sets out some of the potential implications for families with a child affected by a developmental disorder who are undergoing broad genetic testing such as array CGH or exome analysis.  She proposes an increased role for genetic counsellors (GCs) in helping families adapt to genetic test results and proposes a model where GCs hold clinics prior to genetic testing to help manage patient expectations and to identify individual family needs.

 The background overview of the impact of having a child with a developmental disorder, the search for a diagnosis and research into factors that help or hinder adjustment is thought provoking and well structured.  The author should also mention large projects doing whole genome as well as exome analysis for completion (such as 100,000 genome project).

 She sets out well the complex needs of families in these situations and the need to pay attention to emotional needs (counselling) as well as educational needs (teaching) of parents going through testing.  In particular, she highlights the need for management of expectations surrounding a test and a need to signpost/refer parents to additional resources/support groups that may further facilitate adjustment and coping.  Clearly, these families would benefit from incorporation of more of the ‘counselling’ aspect of their consultations in order to optimise their adjustment.

 However, there is more than one way this could be achieved.  Personally I think the idea of introducing a ‘preconsultation’ with a GC prior to broad genetic testing by another clinician (as suggested in the authors model) is a retrograde step for the profession.  I think this would be a great strain on limited GC availability at a time when we are taking steps to deal with increase in referrals by doing more genetic counselling by telephone.  For many genetic services models already exist for pregathering of information by family history questionnaire for certain conditions and some of this is done by coordinators that are not trained in genetic counselling.  This non-GC workforce would not be equipped to ask about family coping strategies or perceived control as seems to be implied in the authors model (under initial contact).

 While it is valid and laudable to highlight GC input and support for families with a new diagnosis or uncertain result, I don’t think this shift in service provision provides the answer.  Rather, any provider (whether Clinical Geneticist or GC) offering a genetic investigation should be equipped to discuss managing expectations and support the family by signposting to resources or offering additional consultations, or referrals as required.  Depending on the centre, It is already common for GCs to see families **after** a ‘genomic’ result to facilitate understanding and assess the families further needs and this could be an open offer or case by case.  Additional training is something that might be required for some providers.  Another part of the solution could be multimedia resources that facilitate the process of testing and follow-up (e.g. online videos about having a genetic test and how it doesn’t always give a clear answer or improved follow-up literature for families).  There is no doubt, however, that, as the author points out, GCs are skilled at managing the psychosocial aspects of genetic counselling and there will be a need to increase the workforce as more complex genetic investigations and results are provided.

 For readers outside clinical genetics services, I think the author should distinguish between the terms genetic counselling and genetic counsellor, as this can be confusing for those in other specialties.  Genetic counselling is done by different types of provider (Clinical Geneticists, GCs and, in some specialties, by specialist nurses who have had genetics training).  Genetic counsellors, however, are a body of allied health professionals from science or nursing background, ‘usually’ not MDs, who are trained in genetic counselling.

 Overall, as this is an opinion piece and raises interesting issues surrounding broad genetic testing. I approve publication with reservation based on a limited exploration or potential solutions and significant reservation about the model proposed.

**Comments by Vicki Wiles, Consultant Genetic Counsellor**

 This review and opinion piece by a Genetic Counsellor from the Wales Genetic Counselling service raises interesting questions about the use of Genetic Counselling resources. The support and counselling issues for families with children with disorders of developmental are well described and the summary of research papers supports the argument that Genetic Counsellors are well trained in counselling skills, with an understanding of genetic concepts and testing and are therefore equipped to help families with children with undiagnosed disorders. I would agree that there is often a need for support that a GC can provide but this support might also be provided by the family Health Visitor, a paediatric Clinical Nurse Specialist or the Consultant Geneticist working with the family.

 I would have liked to see more discussion on how genetic counselling varies across England and the devolved Countries, with an acknowledgement that some services have continued to do pre-clinic work sometimes including home visits, with longer term involvement, while other services have moved to more autonomous working for Genetic Counsellors focussing more on genetic testing where there is known diagnosis in the family. Funding and workforce differences in England have influenced the type of work that genetic counsellor’s focus on and I would have welcomed reference to this. The article would benefit from a broader health resources overview to set the author’s views in context including the financial side to her argument, which might well underpin her views. As genomics moves more into mainstream medicine there will be many, possibly conflicting, demands on genetic counsellor’s time with patients and so this article is timely and merits publication to stimulate debate.

We have read this submission. We believe that we have an appropriate level of expertise to confirm that it is of an acceptable scientific standard, however we have significant reservations, as outlined above.

# Peer review: Referee response for version 1

**DOI:** 10.5256/f1000research.15473.r32695

**Published:** 2018-05-22

**Authors:** Charlotta Ingvoldstad Malmgren, Maria Soller

**Affiliations:** 1 Clinical genetics, Karolinska University Hospital, Stockholm, Sweden; 2 Department of Public Health and Caring Sciences, Uppsala University, Uppsala, Sweden; 3 Clintec, Karolinska University Hospital, Stockholm, Sweden

Thank you for giving me the opportunity to review this manuscript.

 I think that this article highlights several important aspects. New genomic technologies will add to the complexity of genetic disorders. Both pre-test and post-test counselling are of great importance to understand what information the new technologies can bring as well of course the limitations of the tests and results.

 I do also think as the writer states that patients as well as relatives might need even more support related to the results (both when no diagnosis is set or when a very rare diagnosis is found, there is limited knowledge is present).

 It is thus important to be able to help and support the patients in that situation and as the author states in a citation; receiving the result can be not the end but the start of the journey.

 Genetic counsellors’ roles might also change and develop in this genomic area.

 So as a subject I think this is an important paper. However, I have some major issues regarding this paper, which need major revisions prior to possible indexing:

Title

 I think the title is long and not very clear. I think it needs to be shortened, given a more focused topic.

Aim

 For me the aim (focus) of the manuscript is not completely clear. What I can see there are two different focuses:

 1) What support do these parents need regarding acceptance and coping

 2) How can genetic counsellors take part in this area

 I think the manuscript should benefit from either focus on one of those aims OR to clarify that there are 2 aims/focuses.

Structure

 The manuscript should benefit from changing the structure of the text. I think it is a little unstructured moving back and forward between things.

Introduction

 I think the introduction is too long and to detailed on the different definitions on ID. It is not necessary for the aims of the manuscript.

 I would also change the order of the subtitles of the introduction. After the first part on the definition and prevalence I think it would be more relevant of having the section on "Diagnostic Tools". I think that this part also can be shortened.

 Then I think that the section on parental responses and support needs to be put together as one section. There is a bit of overlap between them. Check for overlaps. I do think the section on the different coping issues is very good and relevant.

 At the end of the left column page 3 I would change the focus from "how it will affect the role of genetic counsellors....." to how it would affect the need for support and genetic counselling and if so, how genetic counsellors can participate in this area of support.

Role of genetic counsellors

 I think it is a very interesting approach that GCs can play a role here. However it lacks discussions on what unique skills GCs have to take this role. What is included in GC education? The author talks in a sentence about that GCs need to be more prepared to do this and that the current knowledge is not sufficient and that further education might be needed. I would like to see a development of the discussion on this and references to studies on the education and role of genetic counsellors.

Example

 I do believe that it is good to show things by examples and I think the citations from parents is a good ways of showing parents thoughts and experiences. However, for me the example of childhood overgrowth stands a little on it is own and does not add very much here. I recommend to take that out.

Conclusion

 I think the conclusion could be clearer and should benefit from clarifying the aim/aims of the study.

 In summary, I think it is an interesting paper within an interesting area, but which needs major revision to be indexed.

We have read this submission. We believe that we have an appropriate level of expertise to confirm that it is of an acceptable scientific standard, however we have significant reservations, as outlined above.

# Response

**Published:** 2018-05-25

**Authors:** Flora Joseph

**Affiliations:** All Wales Medical Genetics Service, UK

Thank you very much for reviewing my article. You make some valuable points and I will look to revise the article and incorporate them.
